# Perspectives of older adults, caregivers, and healthcare providers on frailty screening: a qualitative study

**DOI:** 10.1186/s12877-020-1459-6

**Published:** 2020-02-17

**Authors:** Jill Van Damme, Elena Neiterman, Mark Oremus, Kassandra Lemmon, Paul Stolee

**Affiliations:** School of Public Health and Health Systems, 200 University Ave W, Waterloo, ON N2L 3G1 Canada

**Keywords:** Older adults, Frailty, Frailty screening

## Abstract

**Background:**

Screening is an important component of understanding and managing frailty. This study examined older adults’, caregivers’ and healthcare providers’ perspectives on frailty and frailty screening.

**Methods:**

Fourteen older adults and caregivers and 14 healthcare providers completed individual or focus group interviews. Interviews were audio recorded, transcribed verbatim, and analyzed using line-by-line emergent coding techniques and inductive thematic analysis.

**Results:**

The interviews yielded several themes with associated subthemes: definitions and conceptualizations of frailty, perceptions of “frail”, factors contributing to frailty (physical,, cognitive, social, pharmaceutical, nutritional), and frailty screening (current practices, tools in use, limitations, recommendations).

**Conclusion:**

Older adults, caregivers and healthcare providers have similar perspectives regarding frailty; both identified frailty as multi-dimensional and dynamic. Healthcare providers need clear “next steps” to provide meaning to frailty screening practices, which may improve use of frailty-screening tools.

## Background

Frailty has proven to be an evolving concept over the past several decades [1]. The Canadian Frailty Network (CFN) currently defines frailty as *“a state of increased vulnerability, with reduced physical reserve and loss of function across multiple body systems”* [2]. This definition has expanded from a strictly biomedical model [3] to a more comprehensive and dynamic ‘biopsychosocial’ model [1], which includes assets (health, attitudes towards health and health practices, social resources, caregivers) and deficits (illness, disability, disease, dependence on others, burden on caregivers). The dynamic frailty model suggests that changes in either the assets or deficits could influence not only one’s frailty status, but also one’s overall health status [1]. However, researchers remain in disagreement regarding the specific signs and symptoms that should be included in an operational definition of frailty [4].

Putting definition differences aside, there is general agreement that frailty is a state of vulnerability, which is influenced by physical, social, cognitive, economic and behavioural factors [1, 5–10]. Physical factors typically include weight loss, fatigue, decreased grip strength, reduced physical activity, decreased gait speed and disease [6, 7, 9, 11]. Cognitive or psychological factors include cognitive impairment, mental health, attitudes towards health, and spiritual resources [6, 7, 9, 11]. Social factors encompass social resources, social activities, socioeconomic status, loneliness, and social isolation [9, 10, 12]. The relationships between psychosocial factors and frailty are still somewhat misunderstood [10]. However, many researchers agree that factors which extend beyond the bio-medical perspective are important and deserve further attention within academic and clinical realms [10, 13–16].

Research has approximated that 10% of community-dwelling older adults are considered frail and over 41% considered pre-frail [8]. Given the psychosocial component of frailty, inclusion of these components in frailty screening tools should be closely considered [12]. Collard et al. [8] found statistically significant differences in the weighted prevalence rates of frailty when psychosocial factors were included in evaluation. Andrew & Keefe [12] found that for every social deficit, patients experience a 5% increase in mortality risk.

Currently, frailty screening is not considered standard clinical practice within many healthcare systems [17], and thus many individuals who are frail or at risk for frailty may go undetected. Accurate and earlier detection of frailty is important on account of the implications of frailty for individuals, caregivers, and the healthcare system [2]. The healthcare system spends more on individuals living with frailty due to extended stays in acute care and long-term care settings and increased use of community resources and hospital services when compared to their non-frail counterparts [2]. Research has called for targeted and sustained frailty screening to improve individual outcomes [6]. It has been suggested that frailty screening should be implemented for all individuals who come into contact with the healthcare system that are 70 years of age or older [17]. Consistent and targeted frailty screening requires accurate frailty measurement tools that allow for clinicians and researchers to compare the effectiveness of interventions and engage in appropriate care planning initiatives [17]. Previous work has outlined a need to understand frailty from stakeholder and decision maker perspectives [18]. Understanding how frailty screening tools align with the perspectives of the healthcare providers who use them, and older adults and caregivers who rely on their ability to accurately identify frailty, can help uncover any misalignments between research and practice that need to be addressed. For screening to be implemented more routinely [17], healthcare providers must see the benefit; this requires identifying what they feel is important to consider with regards to frailty screening. Similarly, if older adults and caregivers are not being asked about aspects of their life that they feel are applicable to frailty, relevant information may not be as readily shared. Understanding the perspectives of healthcare provider and older adult and caregiver stakeholders can contribute to developing consensus on frailty and facilitate the development of appropriate interventions, policy updates, and knowledge translation initiatives [17].

This study explored stakeholders’ (older adults, caregivers and healthcare providers) perspectives on conceptualizations and definitions of frailty, factors that contribute to frailty, and frailty screening tools.

### Ethical considerations

This study was reviewed and received ethics clearance through the University of Waterloo Office of Research Ethics (ORE #23037). This study had minimal risks associated with it. Risks were associated with participation in group interviews or focus groups, in which we could not guarantee fellow participants would not disclose information that was discussed. To mitigate this potential issue, all participants were asked to keep content of the interviews confidential, and to refrain from discussion regarding the interviews once the interview has been completed.

## Methods

This study utilized a qualitative methodological approach, which is ideal for understanding opinions and perceptions [19], such as those regarding frailty and frailty screening from those who are dealing with frailty on a regular basis. Qualitative data collection - a focus group or individual interviews - were utilized to promote engagement in the study, as some participants were more comfortable in one-on-one settings and others preferred group settings. The lead researcher allowed participants to choose their setting.

This study used triangulation techniques for data collection and included two groups of individuals in the interview sample: older adults and caregivers (OA) (*n* = 14), and healthcare providers (HCP) (*n* = 15). Triangulation permits the groups to be analyzed separately and compared/contrasted to improve credibility and transferability [20]. We treated older adults and caregivers as one cohesive participant group to prevent the stigmatization of older adults who might be frail [18].

This study employed two forms of recruitment. First, purposive recruitment of individuals with some level of familiarity with the construct of frailty was conducted. Second, a snowball approach was used to complement purposive recruitment and expand recruitment pools [21, 22]. As such, participants for this study were recruited from southwestern Ontario through the first author’s personal networks, healthcare clinics, and the Geriatric Health Systems Research Group (GHS) partners such as Seniors Helping as Research Partners (SHARP). While the lead author’s personal networks were utilized, no participants were directly associated with her. Personal networks acted as gatekeepers to reach individuals who would be appropriate for the study. Participants were made aware of the purpose of this project through the letter of information and consent process, and the interviewers introduced themselves to the participants prior to starting to build a rapport.

The individual interviews and focus groups with healthcare providers were completed by the lead author. Focus group interviews with older adults and caregivers were also completed by the lead author who was supported by an experienced researcher who was a member of GHS. Both interviewers had previous experience conducting focus group and individual interviews as part of their academic and professional training.

The focus group and individual interviews followed a semi-structured interview guide, which can be found in supplementary files (see Additional file 3), to explore the conceptualization and definitions of frailty and factors that stakeholders felt were important to include in frailty screening. For example, stakeholders were asked: *“What do you think would make a person or older adult frail?”*. The interview guide was not formally pilot tested but was reviewed by experienced qualitative researchers, co-authors (PS, EN) and a research associate with GHS, as well as an older adult mentoring the lead author to ensure the questions were appropriate. The focus groups and interviews were held at a location convenient and comfortable for participants including private conference rooms at the lead author’s academic institution, healthcare provider’s offices, community centers, or the homes of participants. Focus groups and interviews were audio recorded and transcribed verbatim.

Following Braun & Clarke’s [23] guidelines for inductive thematic analysis of data, the lead author first read and re-read the transcripts to improve familiarity with the content. Second, she utilized NVivo 12 software to develop individual codes using line-by-line emergent coding [23]. Third, she reviewed the initial set of codes upon completion of the coding process to ensure all data were coded, refine the codes when necessary, and improve her familiarity with the data prior to engaging in thematic analysis [23].

Thematic analysis began by collating similar codes together to generate an initial group of themes [23]. This was an iterative process, whereby codes and themes changed and shifted as the researcher continued to read through the transcripts and identify new patterns in the data. After no new patterns emerged from the data, each theme and its associated codes were independently reviewed by the lead author (JVD) and co-author (KL) and potential changes were discussed until a consensus was reached. Thematic analysis was undertaken separately for the OA and HCP groups to permit triangulation.

## Results

Six individual interviews with healthcare providers, one focus group with healthcare providers (*n* = 8), and three focus groups with older adults and caregivers were completed (*n* = 6, 4, and 4) after obtaining informed consent. All interviews were between 30 and 60 min in length, audio recorded and transcribed verbatim. The lead author made written field notes during the interviews for reference during the thematic analysis. Healthcare providers interviewed included physiotherapists (*n* = 2), nurse practitioners (*n* = 5), pharmacist (*n* = 1), physician’s assistant (n = 1), geriatric emergency medicine nurses (n = 2), occupational therapist (n = 1), and geriatrician (n = 1). Healthcare providers practiced in urban and rural locations in south-western Ontario.

Older adults were not asked to disclose if they felt they themselves were “frail” due to the negative connotation and resistance to being labelled as frail that Schoenborn et al. [7] found with their older adult participants. Warmoth et al. [11] found that having older adults self-identify as frail could initiate a “cycle of decline” whereby older adults begin to project their own negative and fearful views of aging onto themselves. Some older adult participants did describe their own perceptions about their frailty status informally during the interviews, but this information has not been included to preserve their confidentiality. Caregivers were individuals who had been, or currently were, involved in caring for a family or friend who they considered frail. They described their experiences caring for a person they considered frail and advocated for what they felt would help their loved one living with frailty.

Participants from each group contributed a unique perspective on the issue of frailty. Recruitment exceeded the original aim of 20 participants - 28 participants (older adults and caregivers *n* = 14, healthcare providers n = 14) were recruited. The anticipated minimum number of participants for each category was based on a study conducted by Monahan and Fisher [24], and supported by research from Hagaman and Wutich [25] that suggests that general qualitative research needs a minimum of 20 participants for relevant themes to be identified and saturation reached. Saturation was identified when no new or emergent information for categorized themes occurred, and no new themes were identified [25]. Saturation, rather than equating with repetition of the same theme defined in the same way, instead indicates that the theme is robust in its description, with rich evidence to support the researcher’s analysis [24, 25]. Using purposive sampling techniques to elicit a wide variety of perspectives aided in reaching saturation, and it is believed that participant views were fully captured.

Three major themes with a direct relationship to frailty screening are described below; a summary table of all identified themes can be found in Additional file 1. The coding tree can be found in Additional file 2.

### Conceptualizations and definitions of frailty

Participants in this study demonstrated that the term frailty continues to lack clarity, with several different conceptualizations of frailty described among participants. Overall, older adults and healthcare providers both described frailty as vulnerability and as multifactorial. One healthcare provider commented, *“I see frailty … as a syndrome of sorts, a condition that … is on a spectrum (participant 12)”* Older adults complemented these sentiments outlining that frailty is “*a condition that one can go in and out of (participant 26)”.* All participants saw frailty as a syndrome highlighted by a lack of flexibility or adaptability to mitigate stressors or handle adversity, and compromise in one or more areas of health.

There was a level of pragmatism in the discussion of frailty among healthcare providers. Providers identified frailty as important to diagnose, with one commenting that this was due to the multifactorial nature of frailty, which increases its significance. Frailty was described by one provider as homeostenosis or reduced reserve capacity:

*“I like that concept of homeostenosis where there is just not a lot of flexibility in the physiologic, social and the whole system. So that person is much more likely to have a decompensation from a particular insult (participant 28).”*

Decomposition and lack of flexibility to adversity was often described by providers as physical deconditioning, withdrawal from activities of interest or pleasure, mental health concerns, or cognitive impairment.

In comparison, older adults described frailty less specifically than healthcare providers, using broad and more generic terms. Their descriptions of frailty helped to highlight the uncertainty in defining the construct. One older adult described frailty as*“not being able to achieve a certain level, be that mental, physical, or anything else (participant 23)”,* while another said that *“you can be frail in one and not another … you could have osteoporosis and have frailty physically, but be mentally just as alert as a tack (participant 7)”.* The older adults and caregivers mostly talked about the physical aspects of frailty, claiming that “*you don’t think about the other aspects (participant 9),”* likely because physical frailty is more easily observable. Older adults also described a dislike for the term frail, indicating there was often a negative connotation associated with the label.

Overall, both groups highlighted that frailty is vulnerability, a reduced ability to adapt to stress, and that it is dynamic in nature. Often frailty was described as triggered by some adverse event such as a fall, illness, death of a loved one, or as stemming from loneliness. Frailty was not considered to be determined by age. One provider noted that they see *“people who are 85 and robust and are not frail whatsoever, and I see people who are 72 or 65 or 60 and are very frail (participant 12)”,* while an older adult commented that *“frailty is not necessarily in elderly or old age (participant 2)”.* These remarks challenge stereotypical views of frailty where age is a key factor in determining a diagnosis of frailty.

### Factors contributing to frailty

Factors contributing to frailty were related to health status. Physical, cognitive, social, pharmaceutical and nutritional factors were each described as being important considerations for frailty status, and are described as sub-themes below.

### Physical factors

Most prominent in the discussion among both participant groups were physical factors. Observable traits associated with frailty such as decreased muscle mass, weight loss, and decreased mobility were described by both groups of participants as they relate to Activities of Daily Living (ADL) and functional capacity. The ability to complete ADLs, or to self-manage, was often associated with strength and mobility by both participant groups. An inability to self-manage was described by older adults as a physical indicator of frailty, particularly when an individual had trouble with bathing or negotiating stairs at home, potentially leading to falls.

Falls were identified by both groups as influencing frailty either by being a triggering event causing frailty or an indication that someone has become frail. Healthcare providers said that hearing that a patient is falling is a worrisome sign which indicates a need for further investigation. One provider commented:

*“I want to know the circumstances of the fall … if they can tell me, you know when, how recent, or has there been multiple, inside [or] outside … was there a pattern? Is there something else going on there that is predisposing you to falling? Are you tripping on your foot or are you blacking out? (participant 12)”*

The circumstances surrounding a fall were used to indicate if an underlying condition related to frailty, such as chronic urinary tract infections or vertigo, needed to be addressed. Alternatively, if a fall caused a fracture such as a hip or leg fracture, then this could result in a new frailty diagnosis due to the impact on ADLs, susceptibility to more falls, or increased risk of infection. Frailty could be prolonged by a continued fear of falling even after a full physical recovery was made. Older adults described falls as a major event, saying that they “*can change everything (participant 25)”* with regard to one’s health status trajectories. Falls were often described as associated with other physical declines, such as vision loss, hearing loss, balance concerns, and declines in energy levels. Some older adults and caregivers suggested that sleep was an important factor for maintaining energy levels, and overall health status. Healthcare providers agreed with older adults that irregular sleep patterns might be troublesome, indicating that napping during the day often resulted in an inability to maintain restful sleep at night.

### Cognitive factors

Cognition was broadly linked to frailty by both healthcare providers and older adults. A prominent cognitive factor that was described by all participants as influencing frailty status was cognitive impairment or dementia. One provider described how the development of cognitive impairment can complicate treatment plans for older adults:

*“It just gets in the way of everything and kind of throws a monkey wrench into any kind of a plan that we would do with prevention. Not completely, but eventually and sort of invariably it can complicate things (participant 28).”*

Cognition was described by one provider as impacting patient motivation, organization and overall ability to complete ADLs, or to appropriately engage in other beneficial interventions such as exercise. Identifying cognitive impairment is important for ensuring that patients are able to follow treatment plans as prescribed and continue to live independently with resources implemented as necessary.

Another prominent cognitive influence on frailty described by both healthcare providers and older adults was mental health status. Providers outlined how depression can influence a person’s drive or motivation to remain engaged in activities of pleasure. One provider described concerns about the mental health status of older adults:

*“Well it is really important … they’re second highest group of depression and they have the means to carry it out. And again, they are losing friends constantly. So, depression is one of the major geriatric giants essentially (participant 28).”*

Older adults felt that mental health is one of the hardest concerns for healthcare providers to diagnose within older adult populations. One older adult commented:

*“ … Probably the hardest for doctors to diagnose [is] mental health. I think one of the most difficult aspects of mental health with regard to seniors are the very subtle areas of so-called age-related issues … loneliness, isolation, abandonment, [and] depression on a relatively low level but chronic, ongoing. That must be [difficult for] doctors to know what to do with. The patient isn’t serious enough psychologically to be sent to a psychiatrist or psychologist and yet they are not recovering (participant 26).”*

Both older adults and healthcare providers identified that depression may be more prevalent due to the chronic losses that are associated with aging. Chronic loss included physical losses such as mobility, vision, or hearing, whereby people are less able to engage with their communities, or as social loss such as death of a loved one, estrangement from family members, or decreased contact with friends and family.

### Social factors

Social loss was described by both participant groups as a significant social factor in frailty risk, and one which could manifest in physical or cognitive symptoms. One older adult described loneliness as influencing frailty through physical manifestations:

“*I think when people are lonely they don’t want to, well they want to interact with other people, but they don’t have the opportunity and then that affects them physically because they sort of sit and vegetate...(participant 9).”*

Both participant groups described how social influences could be the triggering event that initiates the onset of frailty. Older adults identified that communication could become more difficult for frail older adults, and further limit social interactions and feelings of connectedness to those around them. An example described by older adult participants was struggling to communicate through the use of technologies such as telephones due to dexterity issues with dialing a number on a phone, hearing loss, or changes in mobility that make getting up to answer the phone difficult.

Living alone was considered to increase risk of frailty by increasing risk of loneliness, depression, anxiety, or withdrawal from the community. However, healthcare providers acknowledged that living arrangements must align to the values of the patient and ensure their safety. Safety within the home should be discussed by patients and providers. If a patient’s health has declined so that stairs or bathing become risks factors for falls, then resources need to be identified to maintain health status and reduce frailty risk. This requires identification of frailty risk earlier to ensure interventions are received in a timely manner.

### Pharmaceutical factors

Pharmaceutical considerations affect frailty in multiple domains. Providers described the importance of carefully considering prescriptions for older adults due to possible side effects and contraindications with other medications or conditions. One provider described prescribing as a balance of risks and benefits:

*” … everything is a risk/benefit. So, in medication, we can say do you want this medication? But what would happen if we gave the medication? What are the potential side effects? What is going to be the therapeutic benefit? What is the burden of taking a medication?... People don’t want to take pills, or they don’t want to take another pill, so that’s something to consider (participant 28).”*

Providers stressed the importance of thoroughly assessing risks and benefits when prescribing to older adults. Another provider referenced that older adults should not be on any more than five medications, and no more than three medication in one category, describing risks of polypharmacy. Healthcare providers recognized that polypharmacy requires strict adherence and compliance by patients to ensure these side effects and contraindications are minimized. Despite patients taking medications properly, providers described how side effects can still be observed. Pharmacists were identified by older adults as a valued member of the care team, and a resource to help frail older adults manage and understand their medications and associated risks.

### Nutritional factors

Nutrition was described by both older adults and healthcare providers as influencing frailty through multiple domains. Nutritional influences on physical frailty include malnutrition, where patients are not getting the calories nor vitamins and minerals required to maintain good health. Nutritional deficiencies were identified as “*a big part of frailty (participant 28)*” by one provider, and another provider described how malnutrition influences energy levels, perpetuating the risk for falls, inactivity, and cognitive abilities such as concentration. Nutrition was also described within the context of social and cultural norms. Older adults and healthcare providers described social aspects to eating, where individuals associated meals with time for connection. When loneliness or social isolation occurs, providers felt that frail older adults may be less inclined to consume meals. Providers also identified concerns for frail older adults when care transitions are occurring. When a frail person transitions into assisted living or nursing home care, they may not have access to culturally familiar meals, further perpetuating malnutrition. Ensuring older adults have access to familiar foods that provide proper nutrition could improve frailty risk status.

Overall, frailty was described as influenced by physical, cognitive, social, pharmaceutical, and nutritional factors. Healthcare providers and older adults described these factors within the context of influencing frailty risk or frailty status in some capacity.

### Frailty screening

Frailty screening was almost exclusively discussed among healthcare providers. While self-report frailty screening tools exist [26–28], these were not identified nor discussed by older adults. Older adults described factors they felt were important to include in frailty screening, but healthcare providers provided insight into current practices within their healthcare positions.

### Current practices

Providers agreed that identifying frailty was important but felt current literature and screening guidelines were ineffective at articulating the implications of a score. Results should provide more meaningful and action-oriented information for patients and providers. Many described how they used their own methods to determine the functional ability of clients, often based on more formal screens, but modified to suit their unique clinical needs. Providers identified that formal screening tools may be too time consuming to complete, which is why providers’ use of frailty screening was low when not mandated. Even when screening was mandated, providers described an inconsistency in the tools they used within various settings, causing confusion when trying to compare scores from the same individual over time and in different geographic locations. To combat this, many providers described using a comprehensive health history to better understand a patient’s health status and identify when changes have occurred that may need to be addressed. One provider gave an example of why understanding a patient’s history is important:

“*It could be that somebody seems really frail and everyone’s like ‘well he’s 90 this is normal for 90’ [or] ‘Of course, he’s 90!’ And then you find out actually no, this person was working, this person was playing golf, this person was driving a carpool … This is different (participant 28).”*

Health history can also indicate areas of risk. Providers identified lifestyle habits that would influence frailty risk as questions they would ask when discussing health history. These habits included things like smoking, alcohol consumption, and education level.

Home visits were identified by healthcare providers as an opportunity to gather information to assist in assessing a patient. Homecare visits were described as indicating how well a person is functioning within their home through visual observations of the state of the home or how well the person can guide a provider throughout the home. Home visits were also described as giving insights into nutritional concerns by simply looking inside a patient’s fridge or pantry to ensure that what the patient is reporting aligns with what is available within the home. When homecare visits were not possible, providers described using specific questions about mobility within the home, transportation and exercise habits to better understand a person’s lifestyle and routine. Although the answers to these questions are self-reported by patients, they can provide context to a patient’s frailty status, and may identify areas of concern. Providers also described the importance of understanding patients’ self-perceived health status. Providers described comparing their visual assessment with the answers to their formal or informal screening questions, and the perspective of patients to see if these different perspectives align. If there is disconnect between how well a person appears to be doing and how that person feels they are doing, further investigation could be required.

Often current screening practices were described by providers as using clinical judgment. Clinical judgment was referenced as being developed over time with exposure and experience, as well as through mentoring. However, there was disagreement among providers about the appropriateness of using only clinical judgment. Some providers felt that clinical judgment could sometimes override a formal assessment, and lead to further investigation for a patient in spite of the assessment results. Others described clinical judgment as something that should not be relied on, because *“you don’t know what you don’t know (participant 27)”.* Implementing consistent frailty screening could help providers identify concerns related to frailty more effectively.

#### Current tools in use

Several formal frailty screening tools were identified as currently in use during interviews with healthcare providers. These included included the Assessment Urgency Algorithm (AUA) [29] Clinical Frailty Scale (CFS) [5], interRAI tools (interrai.org), Seniors Fitness Test (SFT) [30], short physical performance battery (SPPB) [31] gait speed, sit-to-stands, and balance tests. The CFS was described as easy to use due to the pictures. The visual prompts on the frailty scale make it easier for providers to assign a frailty score. Providers appreciated that the AUA and interRAI tools included a question about caregiver stress as this was described as an important concern. However, providers also articulated concern over the inclusion of the caregiver’s perceptions of a patient’s status in the interRAI as it could introduce bias or inaccurate information depending on the relationship between caregiver and patient. Providers discussed how often family members or caregivers may be out of touch with the actual status of the patient. However, if caregivers and patients have a good relationship and are present in the patient’s life, they can provide helpful information to help direct treatment plans.

The SFT was praised due to the normative data that allowed for easy comparisons, for good test-retest properties, and for the ability to modify the assessment based on the client’s abilities. The SPPB was also used to assess frailty, but providers indicated it was not as sensitive to change as other tools. Standardized gait speed testing was also considered a good indicator of frailty but could be difficult to administer depending on setting. Sit-to-stand testing was considered important to include in assessments as well, as it provided a good indicator of leg strength, and a functional movement pattern. Some providers also added a balance test to assessments, describing it as a good indicator of falls risk, and were surprised it was not included in formal assessments such as the SFT or SPPB.

### Limitations in frailty screening

Providers also described the impact of inconsistent screening. If screening is completed inconsistently, it does not always provide useful or accurate information. One provider outlined how they often get “one-off” screens which make it “*hard to really get an accurate picture … It gives you this picture in this time, but it doesn’t let you know how it got to this point (participant 21)”.* Accuracy was also a concern when discussing what frailty screening tools currently include in evaluation. Providers felt mainly physical aspects of frailty were evaluated in current tools, while other risks commonly observed within their healthcare settings were missing. One provider outlined an example:

“*I keep on getting the one offs … I see and sometimes screen [patients] a six, which is the highest number saying that … they’re not functioning at home. And you look at the person and they are walking and talking, and the reason they’re not functioning at home is because they are sad (participant 21).”*

Providers described how frailty screening tools may be missing important factors such as cognitive, social, and emotional components. These factors were described by both patients and providers as possible underlying causes for frailty, but providers felt they are not captured in frailty tools and so often go unnoticed without further investigation.

### Recommendations for frailty screening

Recommendations for frailty screening were identified by both older adults and healthcare providers. Healthcare providers identified the need for more consistency in how frailty is approached, outlining how “*you’ve got nine different nurses with nine different opinions doing it nine different ways (participant 27)”.* There is a demonstrated need for consistency in the tool that is used and how different tools relate to one-another so results are transferable to different settings. Providers identified the importance of understanding the context in which frailty screening takes place. Many assessments were identified as currently completed in emergency room settings, where an individual is likely already experiencing decreased function and increased vulnerability. Understanding where screening has taken place, the context of why a screen was initiated, and the current state of the patient in this situation, can shed light on the output of the tool.

Providers also outlined a need for consistency in the timing of screening, describing how screening should be completed routinely to identify concerns earlier. Some suggested implementing tools on an annual or even quarterly basis so providers have an opportunity to detect changes earlier. To promote more regular screening, tools also need to be quick and easy to administer. Providers described how “*people don’t pick up on those deficits unless you use the tool (participant 21)”.* Routine screening may use a team approach, requiring better information sharing practices among allied health professionals. Older adults discussed their support for the use of interdisciplinary teams to manage health concerns, describing that many health concerns related to frailty may be better suited to other members of an interdisciplinary care team such as a physiotherapist, nurse, or social worker. This provides promising insights that older adults would likely support using allied health professionals to support frailty management as well. Due to the multidimensional nature of frailty described by both participant groups, multidisciplinary teams provide a good opportunity to screen for frailty based on their area of expertise.

Overall, formal frailty screening was described by providers as needing refinement. Providers identified several areas of concern including accuracy, consistency, and providing meaningful results. Many frailty screening tools were identified by providers, each with benefits and drawbacks. Action-oriented outcomes were described as beneficial for patients and providers and may increase the uptake of frailty screening.

## Discussion

This paper has reported a qualitative investigation of the perceptions of older adults, caregivers and healthcare providers on frailty, and the factors which contribute to frailty, and on the processes and tools used for frailty screening.

### Perceptions of frailty

Older adults primarily discussed the types of factors they felt were important to consider regarding frailty, and healthcare providers expanded on these factors while contributing information about frailty screening processes. Both older adults and healthcare providers identified that frailty was multifactorial and influenced by biological, psychological and social factors, aligning with previous research [9]. In this study, older adults linked frailty to overall health declines and losses of independence. As such, the older adults in our sample disliked the term “frail”, a finding supported by previous research [18]. Warmoth and colleagues [11] found that older adults often resist being labelled as frail because the term ‘frail’ incorporates negative and often fearful views about aging as being “feeble, dependent, and vulnerable”. Older adults who self-identify as frail often use the term to reflect a state of disengagement from activities, both socially and physically, rather than to indicate the presence of functional impairment or poor health [11].

### Frailty screening

Healthcare providers felt nutrition was an important factor to include in frailty screening initiatives because of its influence on other concerns such as fatigue, dizziness, continence, and falls risk. Nutrition is often associated with meal preparation and eating as part of ADLs, and nutritional deficits have been identified in previous work as influencing frailty risk, as they impact overall functional reserve [9].

Falls were described as a possible triggering event for frailty. Older adults placed importance on falls because they were often unable to recover their level of pre-fall functional ability. These sentiments are supported by Ruthig and colleagues [32], who found that fear of falling was founded on elders’ fears about losing autonomy, leading to adverse health consequences. Falls can lead to avoidance behaviours that influence physical activity levels, social engagement, and overall quality of life, which in turn can impact psychological well-being [32]. Perhaps it is for these reasons that providers from all disciplines identified history of falls as one of the biggest indicators of frailty status.

Falling was often linked to polypharmacy. Providers identified polypharmacy as a concern for frail older adults due to the side effects or contraindications often present with consuming multiple medications concurrently. Side effects can include delirium, fatigue, balance concerns, dehydration, increased risk of falls, and increased risk of hospitalization [33]. It is important to understand possible side effects before prescribing, and providers outlined concern over the number of medications older adults consumed, supporting research by Garfinkle and Mangin [34], who describe using a risk to benefit analysis when prescribing medications to older adults to ensure the best outcomes. Older adults were pleased with the larger role pharmacists were playing in care teams, especially with regard to explaining possible side effects to patients. These explanations may help reduce the number of emergency room or family physician visits by making older adults more knowledgeable about what side effects to expect, and by making them better equipped to recognize and handle these side effects. Similarly, if pharmacists continue to be more involved, they can develop pharmaceutical management plans with patients to ensure adherence and compliance. Research has indicated the use of web-based applications and other technologies may support medication compliance through reminders [35].

Cognition has been previously acknowledged as independently associated with frailty [36]. One provider described cognitive impairment as “throwing a monkey-wrench” into self-management plans because you cannot rely on long-term plans or new routines. Cognitive impairment impacts each person uniquely and requires more personalized, flexible and multi-domain care strategies [37]. Cognition also includes mental health status, which participants felt was hard to diagnose, and which can impact health-related behaviours such as motivation to complete ADLs [cooking, cleaning, bathing]. Mental health concerns can affect physical health status and result in an individual withdrawing from activities of pleasure [38, 39].

Identification of mental health concerns may require patients to be more forthcoming with information about how they are feeling, as signs and symptoms can be subtle and require a certain level of trust, disclosure, and vulnerability that can be difficult to muster. Much has changed over the last several decades with the stigma regarding mental health, but stigma continues to influence health decisions [39]. Providers must be aware of the historical and cultural perspectives of patients to ensure they are building a relationship that fosters honest disclosure of information to identify possible indicators of health deterioration.

Social influences on frailty included living arrangements. This study highlighted the importance of a person’s autonomy when choosing living arrangements. As providers identified, some patients wish to move into more supportive living environments, and some choose to age in their current locations. However, when a person’s preferred living environment may no longer be safe, a transition to alternate environments should be supported. Effective care transitions can increase confidence and reduce healthcare utilization [40]. Living alone was considered to increase risk of frailty because it could lead to loneliness, feelings of depression or anxiety, and withdrawal from community engagement. Previous research has identified that living alone is correlated to variables such as depression and marital status, and loneliness has the same associated risk for mortality as other established risk factors such as physical activity, substance abuse, and obesity [41]. Older adults are at a higher risk of loneliness due to the increased number of losses they experience as they age [42]. Loneliness can be a complicated social factor to overcome as it takes time to develop new meaningful relationships with people. As participants identified, loss is a chronic part of aging, and loss of a loved one can be difficult to overcome.

Older adults are currently living longer and healthier lives, and chronological age is not an exclusive determinant of one’s ability to function [43]. Physical appearance, although providing a valuable data point, should not be the determining factor for care. Healthcare providers should strive to maintain health through preventive or proactive care, which can reduce the burden on resources within the healthcare system [43]. Healthier individuals over the age of 70 have lower annual costs within the healthcare system, offsetting any costs due to increased longevity [43]. Frailty, due to its dynamic and multi-factorial nature, is one construct that would benefit from preventive care, and could improve the lives of older adults.

### Current frailty screening methods

Current practices identified several frailty screening tools that were known or currently used in various settings. The CFS was identified as a simple tool whose use of pictures made it easier for providers to implement. The AUA and interRAI tools were currently in use and described as easy to use, but providers were unclear about what the output of the tools meant. Providers wanted to better understand the meaning of these tools’ results and how the results could, or should, influence their care plans. SPPB, SFT, gait speed, sit-to-stand and balance testing were all identified as tools that providers used or adapted to suit the needs of patients and various healthcare settings. Providers liked the normative data that were associated with some tools such as the seniors’ fitness test, as this provided context for both patients and providers on where patients functioned relative to peers. Developing normative datasets for frailty screening tools may help to apply meaning to results for clinicians and improve the use of frailty screening tools across different care settings.

Providers described the use of clinical judgment to ascertain a person’s health status and to decide if further investigation is needed. Clinical judgment was described as a “gut feeling” based on observations that initiated further investigation, sometimes in contradiction to a formal assessment result. This provides interesting insights to providers’ thoughts about screening tools, hinting at a lack of trust in results, and may indicate a need for better training in the implementation of tools and the importance of using screening tools in practice. Providers did express concern over the accuracy of tools currently in use, especially regarding the degree to which the tools are holistic, valid and reliable. Clinical judgment may be useful if tools currently in use are not sensitive enough to detect change, or if a tool does not encompass health holistically. Generally, providers warned that clinical judgment should not be used as a replacement, but rather a method of triangulation or a guide throughout patient interactions.

Frailty screening can be complicated by the screening context. As participants noted, screens completed in emergency situations may not provide an accurate depiction of a person. Similar to a white-coat syndrome, where patients’ blood pressures increase in physicians’ offices [44], frailty scores could fluctuate depending on where the screen takes place. Emergency rooms and hospitals often cause distress for people, and a single screen completed in such a setting is unlikely to provide meaningful results. Routine screening across multiple healthcare settings may be optimal. Further research should look at the accuracy of results across various health settings.

### Strengths and limitations

To improve the methodological rigor of this paper, actions to ensure credibility, dependability, confirmability and transferability were undertaken. Credibility refers to how accurately the data are represented [45] and was ensured by having a second independent researcher review codes and themes, as well as engaging in triangulation of data, where coding and theming perspectives from caregivers and older adults was completed prior and separately from those of healthcare providers. Dependability refers to the reproducibility of the results. This study produced an audit trail regarding decisions made during the research process and detailed methodological processes [45]. This audit trail involves notes on decision processes such as theme development and refinement and was kept in a notebook [45]. Engaging in an audit trail improves reflexivity and helped the authors to clarify decisions before moving forward [46].

Confirmability refers to how objectively the resulting themes and codes match the data and was ensured using inductive, line-by-line emergent coding. This ensured that codes stick as closely to the data as possible, and minimized researchers projecting personal biases onto the data [47]. Lastly, transferability refers to the generalizability of any findings to alternative contexts. To aid transferability, this study utilized interviews with a wide variety of key informants, to ensure that as many possible perspectives as possible were found. Engaging a variety of participants in good interviewing techniques to obtain rich and robust data allowed all perspectives to be considered so that readers relate to the experiences discussed within the subsequent results and discussion sections [45, 48, 49]. Triangulation of data also improved transferability within the context of the research project [20].

The current study has strength in the variety of perspectives that were included in the qualitative analysis. The contribution of older adults, caregivers and healthcare professionals from a variety of backgrounds provided input to frailty and frailty screening procedures across different settings. Furthermore, perspectives were obtained from various parts of Southwestern Ontario, providing input from different geographic locations within the region. There was a large variety of healthcare provider perspectives, but only a few contributors of each type of professional perspective obtained which is a limitation of this study. Future research could examine the differences between providers by profession, and by geographic setting.

Selection bias may have been introduced through recruitment through authors’ personal networks. While personal networks were used, the level of familiarity between the researcher completing the interviews and participants was relatively low. To mitigate any discomfort, researchers outlined that participants were not obligated to answer any questions they were not comfortable with and ensured the deidentification and anonymization of transcripts and quotes used for analysis and this manuscript.

### Future directions

Future work should focus on implementing routine frailty screening in primary care and allied health care settings effectively and efficiently. All stakeholders support interdisciplinary collaboration for frailty, indicating there may be opportunities to utilize these sources of care to improve the effectiveness and efficiency of frailty identification and interventions. As providers discussed, understanding the impact of a frailty score is important for increasing the uptake of routine screening. Future work should focus on clarifying action items for clinicians after a frailty screen has been completed. This may include protocols for what kinds of interventions should be implemented to improve the various factors that contribute to frailty and improving the understanding of how these factors impact frailty risk.

## Conclusion

Overall, older adults and healthcare providers have very similar perspectives of frailty - both understand frailty to be multi-dimensional and dynamic. Understanding each patient as a unique individual may allow for more subtle changes to be observed earlier, and interventions provided sooner. This can be facilitated by more holistic and routine screening processes where changes may be detected earlier. Engaging in holistic screening practices which leave patients feeling more valued as individuals may result in improved patient buy-in, and better adherence to prescribed interventions. However, providers need clarity on what the “next steps” are when completing a frailty screen. This involves improving knowledge on frailty risks and implications which provide meaning to results, and clear action items based on results of frailty screening. Improved clarity on the role of frailty screening tools may improve the uptake of using frailty screening tools across various healthcare settings.

## Supplementary information

**Additional file 1.** Summary of Inducive Thematic Analysis Findings, Identifies and describes themes and provides supporting quotations.

**Additional file 2.** Thematic Coding Trees, Provides the coding trees for healthcare providers and older adults and caregivers that was developed using inductive methods.

**Additional file 3.** Interview Guides, Provides a copy of the interview guides for older adults and caregivers, and healthcare providers, that were used during data collection.

## Data Availability

The datasets used and/or analysed during the current study are available from the corresponding author on reasonable request.

## Abbreviations

ADL: Activities of Daily Living
AUA: Assessment Urgency Algorithm
CFN: Canadian Frailty Network
CFS: Clinical Frailty Scale
HCP: Healthcare providers
OA: Older Adults and caregivers
SFT: Seniors Fitness Test
SPPB: Short Physical Performance Battery
