# Supplementary material for: Perspectives of older adults, caregivers, and healthcare providers on frailty screening: a qualitative study
Source: BMC Geriatr. 2020 Feb 17;20:65. doi: 10.1186/s12877-020-1459-6 (PMC7027098; doi:10.1186/s12877-020-1459-6)
Supplement: Supplementary file 1 — Additional file 1. Summary of Inducive Thematic Analysis Findings, Identifies and describes themes and provides supporting quotations. [file 12877_2020_1459_MOESM1_ESM.pdf]

Additional File 1: Summary of Inductive Thematic Analysis Findings

Theme	Explanation	Quotes
**Conceptualizations and definitions of frailty:**	Frailty was described as a multifactorial, dynamic syndrome that could elicit multiple areas of compromise. Compromise could include physical deconditioning, withdrawal from activities of interest or pleasure, mental health concerns, or cognitive decline. Frailty was described as a change in ability to self-manage due to some kind of compromise.<br><br>Frailty was also described as influenced, but not determined, by age. Stakeholders also identified that frailty is often the result of some triggering event. An event that could trigger the onset of frailty was often described as physical, such as a fall or illness, but could be social or psychological such as loneliness or death of a loved one.<br><br>Frailty was also described as vulnerability, inability to achieve desired level of functioning, and something that could occur in different health domains such as physical, emotional, spiritual, social or cognitively.<br><br>Older adults overall did not appreciate the term "frail". Frail was associated with extreme dependency, end-of-life, physical deterioration, and negative connotations.	HCP: "*You know, that's what makes you frail is that if you have multiple areas of compromise then they can kind of become amplified in the face of a challenge." (p. 28)*<br><br>HCP: *"I like that concept of homeostenosis where there is just not a lot of flexibility in the physiologic, social and the whole system right. So that person is much more likely to have a decompensation from a particular insult." (p.28)*<br><br>HCP: *"I see frailty as a um I mean, I see it as a syndrome of sorts, a condition that yeah is on a spectrum." (p. 12)*<br><br>OA: *"Frail I just wanted to point I think frail is a condition that one can go in an out of" (p.26)*<br><br>HCP: *"I see people who are 85 and robust and are not frail what so ever and I see people who are 72 or 65 or 60 and are very frail adults." (p. 12)*<br><br>OA*: To me frailty is not necessarily in elderly or old age."(p. 23)*<br><br>HCP: *"I think that then predisposes, people become more vulnerable, putting them at greater risk for not being able to live independently, take care of themselves and have a, you know morbid decline."(p. 12)*<br><br>OA: *"Extreme vulnerability because frail can be used in many ways." (p. 26)*

	Often frailty was linked with physical components because it is a visual or observable marker of health.	OA: *"Well basically to me its not being able to achieve a certain level, be that mental, physical, or anything else" (p. 23)*<br><br>OA: *"Ya and you can be frail in one and not another (I: Sure) you know you can have uh from a number of reasons you could have osteoporosis and have frailty physically but be mentally just as alert as a tack." (p. 7)*<br><br>HCP*: "Right so it is usually multifactorial frailty, that's what makes it significant." (p.28)*<br><br>OA*: I think part of the problem is with the definition of frailty. I agree with what [participant] is say about, it's generally, as soon as you say it you think physical. (p. 7) Ya you don't think about the other aspects. (p.9) and also you think negative (p.7)*<br><br>OA: *get rid of the frail term". (p. 10)*<br><br>OA*: "Frail strike me as end of life, frail on your last footing as it were." (p. 26)*
**Physical Factors Contributing to Frailty**	Physical factors contributing to frailty often encompassed functional capacity. This was related to physical strength, mobility, and ability to self-manage. Factors that contribute to one's ability to self-manage, remain mobile, and maintain strength included chronic or primary conditions, injury or pain.<br><br>Falls were also an important consideration to physical frailty. Falls was often considered a "triggering events" that initiates the onset of frailty. Falls were described as a result of physical weakness, vision concerns, balance issues, or some other primary	HCP: *"Well what I generally look at is I look at their physical condition and I'll also look at their primary conditions so do they have diabetes, do they have kidney failure."(p.21)*<br><br>HCP*: "Another one is using changes in mobility. So going from, the person who walks in and then they walk in with a cane and then they walk in with a walker and then they walk in in a wheelchair…But when people transition to a wheelchair...that's a sign that things are going downhill you know."(p. 28)*<br><br>OA*: "It's hard to keep moving when your muscles and joints hurt." (p.2)*<br><br>HCP*: "I think the physical health piece kind of is like an indication of how well someone is able to take care of themselves." (p.22)*

	condition. Falls were thought of as being a major health risk that could change trajectory of health status quickly. Falls could happen when completing everyday activities such as bathing or going up or down steps. The context of a fall was identified as helpful to understanding what next steps should be. Sometimes falls were a result of an acute condition such as a urinary tract infection or a cold.<br><br>Sleep was also identified as being important to consider with frailty. The quality and quantity of sleep can influence fatigue levels, concentration, and other cognitive abilities.	OA*: "Coping with bathing for instance, stepping into a bathtub or even a shower. Negotiating basement steps at home"(p.2)*<br><br>HCP*: "Yeah when I hear that someone is falling I get worried about that."(p.28)*<br><br>OA*: "So yeah absolutely falls risk is a huge thing (OA2: "Oh it's terrible that can change everything" p.25) problems with vision too, tripping over things…"(p.2)*<br><br>HCP*: "I want to know the circumstances of the fall, I get, I do a lot of, if they can tell me, you know when, how recent, or has there been multiple, inside outside … was there a pattern cause is there something else going on there that is predisposing you to falling. Are you tripping on your foot or are you blacking out?" (p. 12)*<br><br>*OA: "a lot of seniors don't get a lot of REM sleep. They sleep, but they wake, they sleep, they wake, they sleep they wake, and they need that REM sleep…and REM sleep is where the body rebuilds itself."(p.7)*<br><br>*HCP: "basically [they were] getting up for meals and then not doing any physical activity during the day and then wanting to go to bed at 8 o'clock at night and sleep right through until 7AM and they are wondering why that is not happening."(p.28)*
**Cognitive Factors Contributing to Frailty**	Cognition was considered a broad factor that contributed to frailty. Cognition was largely linked with mental health status and cognitive impairment. Cognition influences one's ability to organize and self-manage through maintaining routines, engaging with interventions, and having the motivation or cognitive ability to complete tasks of daily living.	HCP*: "It just gets in the way of everything and kind of throws a monkey wrench into any kind of a plan that we would do with prevention. Not completely but eventually and sort of invariably it can complicate things." (p.28)*<br><br>OA*: "There is also um mental frailty as people develop a dementia."(p.9)*

	Mental health was identified as a significant concern across all stakeholders, and described as one of the geriatric giants by health care providers. Depression and anxiety impacts many older adults, and can be perpetuated by increased experiences of loss in the older adult cohorts. Loss in many forms can contribute to mental health status including loss of a person, function, ability, or health. Participants described the importance of identifying these issues, but also how difficult it can be. Identification of more understated mental health concerns may require patients to be more forthcoming with information about how they are feeling as signs and symptoms can be very subtle.	OA*: "sometimes people as they get older they have a tendency towards chronic depression will sink into that depression and that makes them withdraw and furthermore lonely."(p.7)*<br><br>HCP*: "cognition just gets in the way because its that same story about the needing to exercise but not really having the drive and not really having the organization and not really having the recall and the ability to learn to make that happen."(p.28)*<br><br>HCP*: "I guess even just like neurologically our brain depends on habits and routines to keep us doing things and if you think about in older adulthood I mean cognitive decline is not necessarily always the case … but often times that piece is there as well."(p.28)*<br><br>HCP*: Well its really important… they're second highest group of depression and they have the means to carry it out so you always want to, and again they are losing friends constantly so depression is you know one of the major geriatric giants essentially." (p.28)*<br><br>OA*: "All seniors, [loss is] chronic because it's a part it's a natural part of life. No one escapes it. But we are not aware of the depth that it goes to as we get older and it only starts getting out as we get older with the increase in our losses and awareness of our losses. Losses in illness, in physical abilities, they are all loses that create grief and if we cannot identify what it is we are dealing with then it can lead to anxiety, depression." (p.26)*<br><br>OA*: "I think that in general is probably the hardest for doctors to diagnose [is] mental health… I think one of the most difficult aspects of mental health with regard to seniors are the very subtle areas of so called age related issues… loneliness, isolation,* abandonment, *depression on a low level relatively low level but chronic, ongoing, that must be [difficult for]doctors to know what to do with. The patient isn't*

		serious enough psychologically be sent to a psychiatrist or psychologist and yet they are not recovering." (p.26)
**Social Influences on Frailty Status**	Social influences on frailty included living arrangements. Living alone was considered to increase risk of frailty, as it could lead to loneliness, feelings of depression or anxiety, and withdrawal from the community. Living arrangements should, when possible, align with where that person chooses to be. Transitioning into different living situations can cause undue harm when clients are not mentally ready. Living arrangements are often dictated by the safety status of the person, where transitions to new arrangements were necessary to ensure the well-being of the patient. This can be difficult if the cost of living is higher than what patients are able to afford, be that in nursing or assisted living situations, or even their own homes with implementation of homecare.<br><br>Social factors contributing to frailty also included loneliness. Being lonely was linked to depression and anxiety, and could cause individuals to withdrawal resulting in failure to thrive. Loneliness was considered an important factor to look for when assessing overall health and frailty. Loneliness was also affected by loss or change in a partner or loved one. Loss and loneliness was described as a triggering event that could increase frailty status or risk and was linked to cognitive abilities as well.	HCP*: "Um well people who live alone typically are higher risk for um, you know that can be more of a risk factor for frailty then someone who doesn't live alone."(p.12)*<br><br>HCP*: "I think a persons' ability to safely be at home or where they prefer to. I always get a little bit frustrated too when people say everyone wants to stay at home, not everybody does right. So I get frustrated when people say oh no we are going to advocate, nobody wants to go to a nursing home nobody wants to go to a retirement home. Well they do, some people do because they feel that they don't have anyone else. But the person and where they want to be and where their preference is to be to me that's whats more important."(p.27)*<br><br>HCP*: "Now the cost of them staying in these retirement homes is more than people can afford to pay."(p.27)*<br><br>HCP*: "I think I'm going to have this person that has this big problem …because you know they are failure to cope or failure to thrive. When physically they have the capability to do it they just chose not to….And you do see that a lot where people just withdraw right. So then you see the weight loss, you see the unkemptness, you see all those things and its all from one thing and its they chose to do it. (p.21)*<br><br>HCP: *"Prop up that one pillar that's missing so they can complete all the other ones because the other ones aren't the problem it's the one pillar that is the barrier." (p.21)*<br><br>HCP*: "we have clients that just they are lonely, they don't really want to get better or they may be, they just show signs of needing someone to talk to a professional person." (p.11)*

		OA*: I think when people are lonely they don't want to, well they want to interact with other people but they don't have the opportunity uh and then that affects them physically because they sort of sit and vegetate and the less you move, the more physically…or your own spiritual health I think that's important." (p.9)*<br><br>HCP*: "it could be a social insult so if they lose a partner or a partner becomes disabled or something like that or if there is a transition that is another sort of insult."(p.28)*
**Pharmaceutical Influences on Frailty Status**	Polypharmacy was identified, primarily by health care providers, as a factor contributing to frailty. Pharmaceutical interventions were described as needing serious consideration when prescribing for older adults. This was in part due to side effects and contraindications for this age group.<br><br>Polypharmacy was also a concern with regards to adherence and compliance. Taking medications at incorrect times can have unintended side effects. Alternatively, not taking them at all can increase the risks of adverse events like a fall or medical event. Pharmacists were identified as a possible resource to help patients understand medications and possible side effects, but if medications aren't being taken properly they won't always elicit therapeutic benefits. Often providers assume compliance and make changes to care based on these assumptions, when a presenting concern could be due to current medication routines.	HCP*: "But we see it all the time that we see side effect from pills even the ones that they are taking correctly"(p.21)*<br><br>HCP*: "The research that I read is no one should be on more than five meds and you shouldn't have anymore than three meds in one category." (p.21)*<br><br>HCP*:"A lot of everything is a risk/benefit. So in medication, you know we can say do you want this medication but what would happen if we gave the mediation what are the potential side effects? What is going to be the therapeutic benefit? What is the burden of taking a medication you know … People don't want to take pills or they don't want to take another pill, so that's something to consider." (p.28)*<br><br>OA*: "I really like that pharmacists are more involved now in medications…they take much more time telling you "okay now this is going ya know, its got side effects" (p.5)*<br><br>HCP *:"if we assume that someone is adhering to something than we are going to make changes based on that but if a person is not even taking the medication to begin with, increasing the dose isn't going to be helpful."(p.28)*

**Nutritional influences on Frailty**	Nutrition was linked to several aspects of frailty included social, physical, and cognitive. Nutritional influences on physical frailty included malnutrition, hydration, continence issues and weight loss.<br><br>Health care providers also linked nutritional issues to energy levels or fatigue, which influences not only a person's ability to concentrate but other cognitive abilities as well. Nutrition also linked to social aspects and the rituals of eating. With decreased palates older may not enjoy eating the same way, or if they have moved they may not have access to the kinds of foods that are important to them.	HCP*: "I mean I think nutritional deficiencies are a big part of frailty" (p.28)*<br><br>OA*: "…not eating, malnutrition, not eating properly."(p.6)*<br><br>HCP*: "Well for energy one, if they aren't eating well, taking in enough fluids they are not going to, they are going to complain of even more fatigue."(p.11)*<br><br>HCP*: "Chances are if they don't eat enough too, I mean quite commonly people are struggling with constipation, they aren't push food through..."(p.11)*<br><br>HCP*: "You know, if they've lost more than five percent of body weight, kind of in the last five years that's a flag to me that you know somethings going on."(p.11)*<br><br>HCP*: "the social piece whether they live* alone *or not can certainly tie in with um yeah the nutritional piece."(p.11)*<br><br>HCP*: "You know and a lot of it is people want to eat their cultural foods too right. If something changes and they're not getting their usual noodles or whatever then that could be a problem." (p.28)*
**Frailty Screening: Current Practices**	Providers described their current practices when meeting new patients within their roles. Many described how they use their own methods to determine the functional ability of clients, often based on more formal screens, but modified to suit their unique situational needs. Providers also identified that formal screening tools may be too time consuming to complete, which is why uptake is low when not mandated.	HCP*: "we do ask questions about how they get around in their home…How do they get along in their home, do they have grab bars, do they have access to transportation, what do they do for exercise? They basically have a functional level scoring when they are assessed by myself and their anywhere from very low to high functioning. So I put them in a category, the very low um clients are ones that I'm going to follow a little more closely because they just don't have access…but I don't necessarily assess them in any kind of survey."(p.12)*

	Providers also described how helpful a home visit can be to accurately assess a patient. Homecare visits can often indicate how well a person is functioning within their own home through visual observations of the state of the home and how the person can guide a provider throughout the home. Home visits can also give insights into nutritional concerns by simply looking inside a patient's fridge.<br><br>Providers also identified the importance of understanding how their patients perceive their own health status. This can act as a method of triangulation, comparing their visual assessment, with the answers to their formal or informal screening questions, with the perspective of the patient. If there is a disconnect between how well a person appears to be doing and how that person feels they are doing, further investigation could be required.<br><br>A large part of how providers currently assess patients is through a patient history. Understanding a patient's history can indicate when a change has occurred that may need addressing. Health history can also indicate areas of risk. Providers identified lifestyle habits that would influence frailty risk as questions they would ask when discussing health history. These habits included things like smoking, alcohol consumption, and education levels as well.<br><br>Often current screening practices involves the use of clinical judgement. Clinical judgement was referenced as being developed over time with exposure and experience, as well as through	HCP*: "Uh I mean I'll ask them but I also, usually say do you mind if I have a look in your fridge? Um yeah cause I've looked in fridges and there is almost nothing in the fridge whatsoever. And if they say they make their own meals, um okay so what might they, maybe they are making some instant stuff." (p.12)*<br><br>HCP*: "I want to know their perspective of how their doing in their own home you know are they independent with their ADLs, their IADLs and as part of that piece"(p.27)*<br><br>HCP: "*I mean starting with uh things like just you know taking a history and looking at their environment…"(p.12)*<br><br>HCP: "*Or it could be that somebody's really seems really frail and everyone's like well he's 90 this is normal for 90 of course he's 90 and then you find out actually no this person was working, this person was playing golf, this person was driving a carpool, you know like this is different."(p.28)*<br><br>HCP*: "If they smoke, if they have a lower education, so those are just kind of some things I might ask about" (p.12)*<br><br>HCP*: "When you've been working long enough and you've seen enough people over the years, you can sense it. No not sense it that's not the right word. But you are observing things that are, you know they are going to be at risk for in the future."(p.22)*<br><br>HCP*: "You can't take the short cuts…you don't know what you don't know."(p.27)*

	mentoring. Some providers felt that clinical judgement could sometimes override a formal assessment, and lead to further investigation for a patient in spite of results, but was also described as something that should not be relied on.	
**Frailty screening: Current Practices (tools in use)**	Current screening tools identified during interviews included the Assessment Urgency Algorithm (AUA), Clinical Frailty Scale(CFS) InterRAI tools, seniors fitness test (SFT), short physical performance battery (SPPB), gait speed, sit-to-stands, and balance tests.<br><br>Providers liked that the AUA included a question about caregiver stress as this was described as an important concern. The CFS was described as easy to use due to the pictures. Providers had conflicting feelings about interRAI tools; with some describing concerns about how subjective, the tool was while others voiced appreciation over the inclusion of caregiver stress. Providers also voiced concern over the inclusion of caregiver's perception of patient's status, which could introduce bias or inaccurate information. Providers discussed how often family members or caregivers may be out of touch with the actual status of the patient.<br><br>Tools that looked more obviously at physical components, such as the SFT, were praised due to the normative data that allowed for easy comparisons. The SFT was also described as a positive tool for test-retest scenarios to observe if interventions were effective, and could be modified to suit the patient's abilities. The SPPB was also used to assess frailty, but providers indicated it was not as sensitive to	HCP*: "I mean that's one thing I would say about the AUA is it asks about caregiver, how stressed are they? You know. That's an important question." (p.28)*<br><br>HCP*: "Well I find [the CFS] easy to use because it gives you those descriptors and it even has the little picture so its pretty easy to put someone on a frailty scale I think." (p.28)*<br><br>HCP: "*Um well the one thing that I don't really like about [interRAI] is there is questions about the family members, about their perceptions. I think it is a good thing but its not a great thing because again a lot of times you get family members that disconnected."(p.21)*<br><br>HCP*: "You know what I mean the RAI is perception. So you're going to ask them how they perceive them, and then I'm going to put in my note how I perceive them with my physical assessment skills… I mean I look at every little thing to say every little key that I can think of when I see you to see how you're managing at home. So if you've got like really wrinkled clothes on and you tell me that you're in the best of shape and your hair is all standing up and you smell like pee and poo I'm going to be like eeeee."(p.21)*<br><br>HCP*: "So [the SFT] is what I use for their baseline fitness level, and that's what I'll retest when their finished their six months with us."(p.11)*<br><br>HCP*: "I look at a six minute walk test. If they can't do a six-minute walk test they can do a two-minute walk test. So senior fitness test uses the six-*

	change as other tools. Standardized gait speed testing was also considered a good indicator of frailty, but could be difficult to administer depending on setting. Sit-to-stand testing was considered important to include in assessments as well, as it provided a good indicator of leg strength, and a functional movement pattern. Some providers also added a balance test to assessments, describing it as a good indicator of falls risk. Providers have identified falls risk as a contributing factor to frailty, and one provider voiced surprise that it was not included in formal assessments like the SFT or SPPB.	*minute walk test but I might modify that and do a two minute walk test if they just cant" (p.11)*<br><br>HCP*: "I use [the SPPB] to help me figure out where they should exercise, but I find I have originally retested clients when I started using it and I find its not extremely sensitive to change."(p.11)*<br><br>HCP*: "If I can do a standardized gait speed test I'll do that depending on if I'm in a house I often can't cause there isn't a stretch that I can accurately do an objective test."(p.11)*<br><br>HCP*: "Up and down from a chair five times can you do that? Can you even do it once? You know without using your arms? I mean that's a huge one right indicating lower extremity strength…"(p.11)*<br><br>HCP*: "Because [balance is] a very important tool, it's a very important sorry, thing to assess because, and I'm really surprised that its not part of the senior fitness test, but it is definitely an indicator of fall risk."(p.12)*
**Limitations in Frailty Screening**	Provider's main concern with frailty screening was a lack of understanding about what the results mean. Providers agree that identifying frailty was important, but screening is ineffective at articulating the implications of a score. Results should provide more meaningful information for patients.<br><br>Providers also described the impact of inconsistent screening. If screening is completed inconsistently, it does not always provide useful or accurate information.<br><br>Compliance or consistency in frailty screening was considered another area of concern. Providers	HCP*: "I think in summary really, I think its an area that's important but I guess I can't repeat it enough times frailty is important you can identify but its what are you going to do with the information that you gain." (p.27)*<br><br>HCP*: "[They should be] meaningful for the patient, not just for the hospital, not just for the regulatory body, for the patient. Like why are we doing all of this?"(p.28)*<br><br>HCP*: "Again when you're doing, as I like to call it one offs, its hard to really get an accurate picture. It gives you a snap shot but it doesn't give you the video so to speak. It gives you this picture in this time but it doesn't let you know how it got to this point."* (p. 21)

	discussed how there is no consensus on which tool is best, and as a result if screening is even implemented in a health care setting they are not always using the same tool. This can complicate things when a person moves or is referred to a different clinic. Consistency within institutions was also identified as a concern, as currently there is no real consequence for not completing screening. Providers also describe how compliance in screening does not always mean patients are receiving the best care. Some providers felt that the time it took to assess a persons frailty risk is time that could have been used more effectively helping patients.<br><br>Providers identified concerns with the accuracy of current screening tools as well. As screening is most consistently completed in emergency settings where patients often feel more vulnerable, providers were unclear how accurate an output the tool would provide. Accuracy was also a concern when discussing what frailty screening tools currently include in evaluation. Providers felt mainly physical aspects of frailty were evaluated in current tools, missing other risks commonly observed within their health care settings. In particular, providers felt cognitive, social, and emotional components were often missed in current tools.	HCP*: "Some hospitals do it, some hospitals don't. So then again you have the problem whereas or if they come from a different region."(p.21)*<br><br>HCP*: "And [institutions] are never going to put as we call it teeth behind to make you do it. Like so if you don't do your normal assessments like your vital signs or something, you're going to get a talking to because they have a computer that check up on you…this person didn't have vital signs for you know 12 hours and their a level two. You know what I mean, that stuff is all in the computer and they can just look right away. I: Right and that is going to have some consequences … HCP: Oh absolutely" (p.21)*<br><br>HCP*: "Yeah and then some auditor comes by and says oh your compliance with screening is 90%. Like that's great (I: Great but what does that) but if yeah. But instead of doing that if you could've spent that three minutes saying you know what can I do to help and really saying you know just really finding out what's important. I don't know."(p.21)*<br><br>HCP: "*I keep on getting the one offs…I see and sometimes screen them a six which is the highest number saying that they basically can't, they're not functioning at home (I: Right) and you look at the person and they are walking and talking and the reason their not functioning at home is because they are sad." (p.21)*<br><br>HCP*: "Well I don't think they ask about, I don't think they really pull in a lot of the, they don't pull in a lot of what I see the cognitive risks, social risks, that kind of, what I would call the psychosocial stuff."(p.11)*<br><br>HCP*: "It is certainly missing though, in my opinion, the cognitive, emotional component, which is a big deal. It's a huge deal."(p.12)*

**Recommendations for Frailty Screening**	Providers outlined several recommendations for frailty screening, mainly the need for more consistency. Consistency is required in how frailty is approached, the tool used, the timing of screening, and what any next steps would encompass. Similarly, tools that are used must have good inter-rater reliability to ensure bias is reduced and an accurate representation of patients is produced. Tools need to be quick and easy to administer, which may help compliance and uptake of their use. Providers described how you often can't identify problems if you don't have a tool to track them.<br><br>The timing of screening was another big recommendation. Providers felt that screening should be completed routinely to better identify concerns earlier. Routine screening may use a team approach, requiring better information sharing practices among allied health professionals. Older adults discussed their support for the use of interdisciplinary teams within their health. Older adults also identified that many health concerns related to frailty may be better suited to other members of a care team such as a physiotherapist, nurse, or social worker. This provides promising insights that older adults would likely support using allied health professionals to support frailty management as well.<br><br>Providers identified the importance of understanding the context in which frailty screening takes place. Many assessments are currently completed in emergency room settings, where an individual is likely already experiencing decreased function.	HCP*: "Right, so…trying to figure a way that you could train or provide the structure or the support so that those things could be done more consistently. But you're still going to have, because it's a subjective measure you're still going to have some variances"(p.21)*<br><br>HCP*: "But the problem you've got, I think we've got nine different nurses, so you've got nine different nurses with nine different opinions doing it nine different ways."(p.27)*<br><br>HCP*: "…if there was one that was say, to the point. Not brief but, not overly (I: Extensive?) yeah."(p.11)*<br><br>HCP*: "And the problem is people don't pick up on those deficits unless you use the tool." (p.21)*<br><br>HCP*: "It should be done on a Q4 month basis or Q3 month basis because it is a very limited amount of time and what it does is it gives us a baseline…"(p.21)*<br><br>HCP: *"And it would be nice to say oh I see she scored a three here she scored a four here she scored a five here she scored a six here look at the progression. What did we miss?"(p.21)*<br><br>OA*: "this is where, you know, these clinics that some doctors have set up, are an excellent idea. Because you've got a dietician, you've got a physiotherapists, you've got an occupational therapist, you know you've got all these people, and so you know the doctor can call on all these people for extra assistance." (p.9)*<br><br>OA*: it seems to me that a lot of this is more ideally suited to one or two other members of the care team, be it a nurse a social worker or whatever… you can't just ignore the doctor, he's at least going to have to push it out to somebody in that instance." (p.23)*

	Understanding where screening has taken place, the context of why a screen was initiated, and understanding the current state of the patient in this situation can shed light on the output of the tool.	HCP*: "A person is going to look a little bit frailer in the emergency department when they come in if it's a medical condition that brought them in."(p.27)*
**Frailty Screening: Recommendations for Frailty Screening (Factors that should be included in screening)**	Providers identified the importance of having a baseline for screening. Baseline screening can give insights as to which facets of health providers should be paying more attention to. Providers described the importance of having more holistic tools to monitor frailty in order to provide patients with the appropriate type of resources and support required to thrive on their own again.<br><br>Providers discussed the importance of assessing functional abilities such as mobility, strength, and level of independence. Pain was also identified as important, as it can often be a limiting factor in mobility and level of independence.<br><br>Pharmacy was considered an important aspect that should be included in frailty screening as well. Providers discussed the importance of assessing the risks and benefits of prescribed medications, as many have adverse side effects and contraindications for older adults.<br><br>Nutrition was also identified as important to include in frailty screening. Nutritional deficiencies and reduced caloric intake have been linked to unintentional weight loss, fatigue, and even falls risks.	HCP*: "So if you are going to support them, you're going to have to be addressing different things and you should be evaluating it at the beginning so you know what the concerns are uh things you need to be paying attention to (I: what the baseline is) yeah what the baseline is showing for this person."(p.12)*<br><br>HCP*: "Right, so its providing the support in a way to support that person socially or emotionally or psychologically so that they are able to thrive on their own again" (p.21)*<br><br>HCP*: "Okay well I'd say for mobility stuff I'd definitely be doing um gait speed and like a timed sit to stand. Those would be big ones I would be doing and grip strength. But for sure the first two. And is that for overall strength, you know lower extremity strength is linked to your ability to lift independently, you know that type of thing."(p.12)*<br><br>HCP*: "Pain, if they have any pain, I mean I always ask about if there is any discomfort that is limiting you…" (p.12)*<br><br>HCP*: "A lot of medication recommendations though, I think in people who are frail is a risk/benefit." (p.28)*<br><br>HCP*: "So when I get into an objective assessment, you know I ask about their weight cause that's something I certainly will pick up on and be aa a flag for me so has your weight been stable, has your weight changed in the last year, if it has how much has it changed?" (p.12)*

	Psychological aspects of health, such as mood, mental health, feeling safe in environments, and cognitive abilities were also described as important for frailty screening. Providers described frustration at a lack of cognitive screening currently being completed, particularly as cognition can influence health in many different ways.	HCP*: "one of the things that frustrates me is when there is no cognitive screening…I'm big on cognitive screening…I don't care if they're here for a non-cognitive reason. I want to know what their cognition is like because maybe they are here because their falling and maybe that's because a person is taking a blood pressure pill twice a day instead of once a day and maybe that's because they have dementia." (p.28)*
**Providers Experience with Frail Older Adults**	Providers discussed experiences with frail older adults within their practice. Providers all identified having frail older adult patients, but one provider commented that when they started to screen more routinely, patients were not as frail as what they had assumed. However, providers discussed that patients often over-estimate their abilities. Older adults discussed this as hiding certain health related concerns from providers. Providers identified that one way they felt older adults did this was through the use of humour. Humour was often used to cover up concerns, providing a response to the question asked without providing a real answer.<br><br>Providers also described how patients could get defensive about their health status. Providers used strategies to build trust to facilitate more honest and forthcoming responses. Older adults supported this, commented on the importance of building a trusting relationship with providers. Many providers believed this defensiveness was out of fear. Providers felt that older adults were fearful of being forced to do something they weren't ready to, like move to new living arrangements, or stop activities they enjoy.	HCP*: "Cause we kind of found, I actually remember when we were gathering everybody like how many were frail I was kind of surprised at how many…I'm not seeing people that are as frail as I think they are but that was an average" (p.28)*<br><br>HCP*: "Functionally looked great, had a sense of humor [that] covered everything up."(p.27)*<br><br>OA*: "It seems like a lot of the issues come from not wanting to admit somethings wrong." (p.8)*<br><br>HCP*: "I think probably more probing. What I found is again if you get into a certain structure in how you ask questions I kind of gage when I see somebody whether their going to be as forthcoming or not"(p.27)*<br><br>OA*: "And make them feel comfortable with you and they will start sharing." (p.25)*<br><br>HCP*: "They don't like to share and much of that is because they don't want you to know that they're not doing well for fear of going into a home."(p.27)*<br><br>OA*: "And there are also a lot of people who are afraid to go into anything else because a lot of situations are not good..."(p.7)*

	Providers also identified their role in providing information to older adults. Older adults often asked providers about how their health status compared to their peers, but also about their current conditions. Many wished to understand the prognosis and what to expect, and how to best plan ahead for what may come. Older adults described this as well, identifying their desire to understand the progression of their diagnosis. Many described the importance discussing the resources that are available with providers, as many are unfamiliar. Building a positive and trusting relationship between patient and provider is important for successful treatment and management.	HCP*: "kind of wanting to know where they are at yeah compared to other seniors in their age group. Like is this normal, is this worse? You know what can I expect? I think those are the broad things"* (p.28)<br><br>OA*: "you want to know sort of how it would affect your physical health and how it would progress that you would maybe ugh, you'd want to like do, manage things for yourself as long as you could." (p.1)*<br><br>HCP*: "they want to know where they are at like what stage they are at, and then they want to know how to plan ahead to an extent."(p.28)*<br><br>OA*: "Well and also* to know that these things are available. A lot of *seniors … just are not familiar… if you're not aware of all the services…".(p.6)*
**Routine and its Influences on Frailty**	Routine, and more specifically a change in routine, was described as a way to identify frailty related concerns. Routine was described as changing when health concerns arise, and this can help provide context to concerns such as sleep, nutrition, mobility, and the overall engagement on an individual.<br><br>Routine can indicate how well a person is managing their day-to-day lives including the management of other factors identified as influencing frailty risk. Routine can indicate how well a person manages finances, medications, and social engagements.<br><br>Routine was identified as giving older adults purpose, and deviation from routine may indicate cognitive or other mental health concerns. Routine ensured that patients are managing various aspects of their own	HCP*: "she was like pretty robust, strong lady but because of her vision loss she isn't able to cook as much its difficult for her. Because of that she doesn't mobilize as much and then shes getting older and there is sickness and what not that come into play. So something like that right its important to know because that impacts (I: All sorts of thing) how one does all their day-to-day occupations." (p.22)*<br><br>HCP*: "they are often housekeeping type things like exercise, socialization, food security, planning ahead like all that kind of stuff, seeing a doctor, seeing a nurse you know. And then the productivity piece is like how are they cleaning their homes, how are they getting around in the community, cooking meals, preparing meals, managing their medication, managing their finances." (p.28)*<br><br>HCP*: "basically [they were] getting up for meals and then not doing any physical activity during the day and then wanting to go to bed at 8 o'clock* at night and sleep right through until 7AM and they are *wondering why that is not happening."(p.28)*

	lives. It can provide broader context to older adult concerns.<br><br>Routine also includes understanding a person's communication style, and frequency of communication with others. Communication can influence the support network older adults have, and issues may go unnoticed by family, friends, or healthcare providers if individuals don't have the capacity to communicate effectively.	HCP*: "Whether its something like for example brushing your teeth and showering and what not. Its not something, it can bring enjoyment to our lives but more so we do it because it has to get done and then once those things are done it allows us space to do other things in our life that are enjoyable and meaningful to us."(p.22)*<br><br>OA*: "and also when you're frail like, communicating with other people whether its family or friends, like uhm, well like say on a telephone… (I: can become more difficult?) OA: yeah."* (p.1)
**Frailty Implications and Risks**	Frailty was described as influencing overall health status, often related to more complex health concerns. Participants identified that when health status has dropped, it is difficult to get back to the level of functioning they were at beforehand. A decrease in health status could be due to surgery or other strenuous modalities, or a triggering event like an illness or fall.<br><br>Recognizing the impact that surgery, illness, or falls may have on health status can influence patients choices for the types of interventions they would like to engage with. Some felt that living with their current condition was safer than risking surgery and the implications that can have on health status. Another described how some patients are just ready to be in a wheelchair and don't wish to work towards using a walker or cane.<br><br>Frailty was also described with regards to its influence on mental health and psychological concerns. Frailty can perpetuate the mind-body link with concerns like depression, where someone may	HCP*: "Yeah and then the other factor that you find is as frailty goes up the common medical conditions that you've lived with all your life become worse..."(p.21)*<br><br>HCP*: "studies show that when you have that drop off most people don't get back to even the level that they were at."* (p.21)<br><br>OA*: "they don't recommend I understand…because they know that its no picnic when you… have surgery and when you're over 80 your recovery is a lot slower if at all so yeah know…ugh you might be better off just living with what you have."* (p.5)<br><br>HCP*: "[Family] want them to you know get out of the wheel chair and start walking again…which is a great goal absolutely that would reduce their frailty levels but in order to, for some frail individuals especially with cognitive impairment, in order to have a sustained upgrade of mobility it would mean a lot of work. Maybe that person what they are really telling you is 'I'm ready for the wheelchair. You know like I don't want to do the work like I'd prefer to do other things'."* (p.28)<br><br>HCP*: "Lets say for example there is a frail older adult with a co-occurring diagnosis of depression or some type of mood disorder that in itself is a barrier but the frailty can just be another barrier to add to that*

	not have the ability to be more mobile, and is also struggling mentally to find motivation to engage in positive health behaviours.<br><br>Older adults also linked frailty to an increased risk of elder abuse. Abuse was described as a serious issue that may not be reported accurately as participants described how it can be hard to identify due to cognitive impairment or side effects of pharmaceuticals on judgment and memory.<br><br>Participants described a need to be more proactive in frailty prevention to mitigate some of these risks. Providers described a desire to identify frailty and associated risks earlier, and treat concerns collaboratively with patients and other providers are appropriate. Preventative medicine was described as a cost-savings, saving the health care system money in the long term, but needing an influx in funding to start this process may prove difficult.	*or a challenge um where someone has difficulty for example like picking up their groceries and cooking in addition to like lacking the initiation or wanting to do that."(p.22)*<br><br>OA*: "One of the risks when people are frail I think too is senior abuse (agreement) it's a big issue."(p.26)*<br><br>HCP*: "So again proactive my famous term is being proactive not reactive because anytime you're reactive you've already lost because you're behind." (p.21)*<br><br>HCP*: "frailty is just another one now that is getting a lot more recognition of the importance of kind of paying attention to maybe early warning signs, early red flags and planning and bringing in multiple disciplines to manage them." (p.12)*<br><br>HCP: "*But it's a problem in medicine that we don't do a good job of. We don't do a good job at the preventative medicine that could save us a whole host of money." (p.21)*<br><br>OA*: "because if you can keep seniors healthy longer its much cheaper for the health care system (yeah, that's true) plus the fact that then we're a happier lot! (laughter)"(p.9)*
