# Supplementary figures and images for: Perspectives of older adults, caregivers, and healthcare providers on frailty screening: a qualitative study
Source: BMC Geriatr. 2020 Feb 17;20:65. doi: 10.1186/s12877-020-1459-6 (PMC7027098; doi:10.1186/s12877-020-1459-6)

## Older Adult and Caregiver Coding Tree

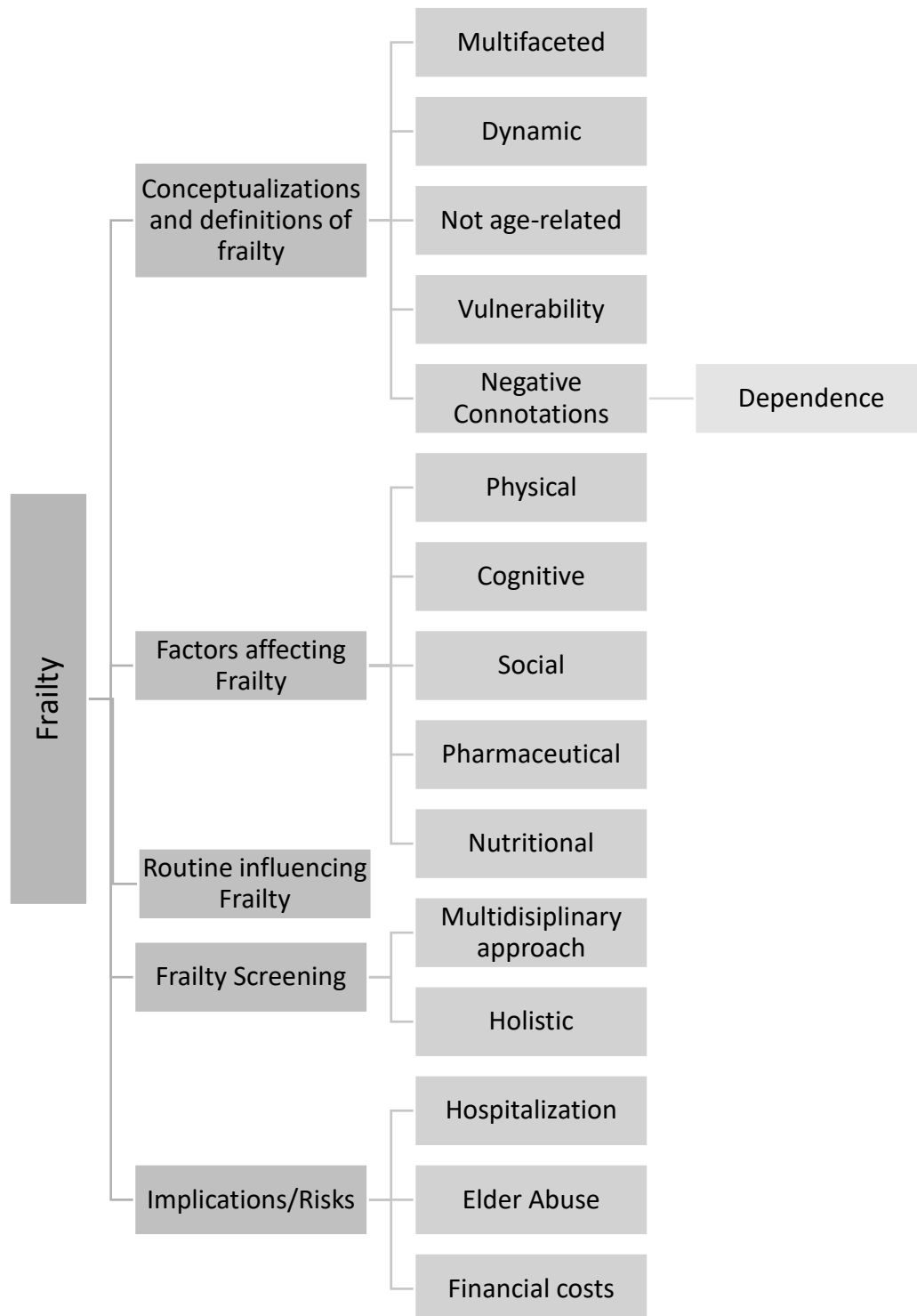

## Healthcare Provider Coding Tree: Frailty

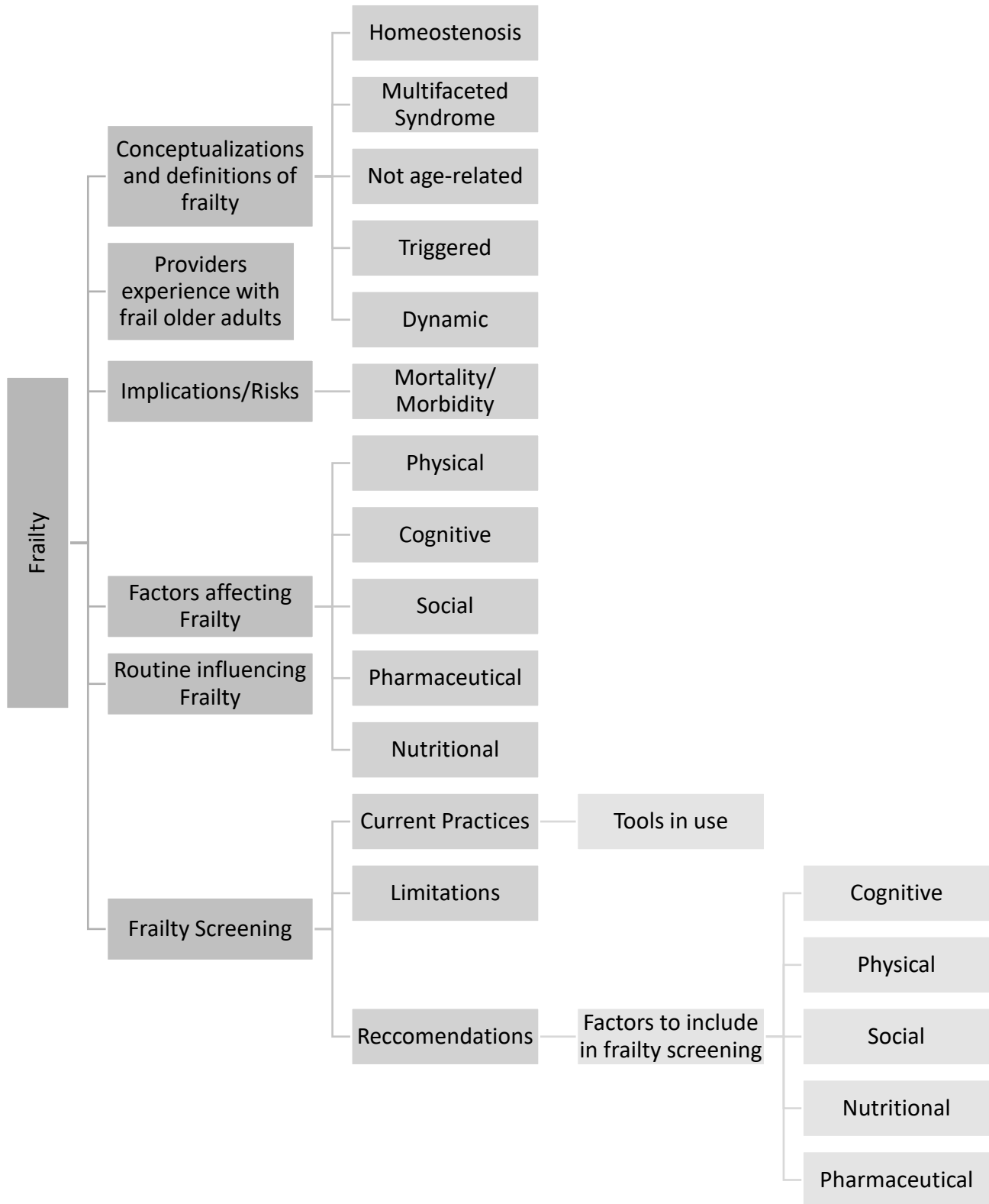

Supplement: Supplementary file 2 — Additional file 2. Thematic Coding Trees, Provides the coding trees for healthcare providers and older adults and caregivers that was developed using inductive methods. [file 12877_2020_1459_MOESM2_ESM.pdf]
