# Supplementary material for: Perspectives of older adults, caregivers, and healthcare providers on frailty screening: a qualitative study
Source: BMC Geriatr. 2020 Feb 17;20:65. doi: 10.1186/s12877-020-1459-6 (PMC7027098; doi:10.1186/s12877-020-1459-6)
Supplement: Supplementary file 3 — Additional file 3. Interview Guides, Provides a copy of the interview guides for older adults and caregivers, and healthcare providers, that were used during data collection. [file 12877_2020_1459_MOESM3_ESM.pdf]

Introduction Script- Interview Guide: Care Givers and Older Adults

Summary of Project: Many older adults are considered "frail" or at risk for poor health. A number of tools or methods exist to identify frailty, but it is unclear if these tools consider what is important to older adults, and as such, the services provided may not meet their needs. This study involves a review of the current literature to determine what health factors are currently included in screening tools that help to identify older adults at risk for negative health outcomes. I am interested in screening that is done within primary care settings (e.g. care that is provided by your family doctor and other health care professionals). I am hoping to have conversations to understand what older adults, caregivers, and health care providers feel should be asked about, or screened for, with regards to older adults health and quality of life. I hope to compare what screening tools currently consider to what older adults, caregivers, and health care providers actually feel is important.

Thank you for taking the time to participate in this study. I wanted remind you that you may choose not to answer any of the questions and can end your participation at any time during this interview. Your confidentiality will be respected and your name will not be associated with the data. We will be audio-recording this session. Please let me know if there are any questions before we begin.

1. What are some topics you feel are important to discuss with health care providers related to health or quality of life?
    a) Probe: medications, health conditions, social supports, recreational activities

2. Why do you feel these topics are important to discuss with your primary care provider?

3. How often do your care providers initiate conversations that capture a variety of these topics and important issues?
    a. What topics or conversations happen most often with your providers ?
    b. What do you wish your health care providers would ask more about/or focus on more closely?

4. What does the word "Frail" or "being frail" mean to you?
    a. Probe: How would you describe frailty?

5. What things do you think would make a person or older adult frail?
    a. Probe: What signs or symptoms would you say contribute to frailty?

6. How do you feel these factors should be monitored to reduce or prevent frailty in older adults?

7. Is there anything that I haven't asked about that you feel is important to share related to our discussion today?

Introduction Script – Interview Guide: Health Care Providers

Summary of Project: Many older adults are considered “frail” or at risk for poor health. A number of tools or methods exist to identify frailty, but it is unclear if these tools consider what is important to older adults, caregivers and health care providers, and as such, services provided may not meet their needs. This study involves a review of the current literature to determine what health factors are currently included in screening tools that help to identify older adults at risk for frailty. I am hoping to have conversations to understand what older adults, caregivers, and health care providers feel should be asked about, or screened for, with regards to older adults health and quality of life. I hope to compare what screening tools currently consider to what older adults, caregivers, and health care providers actually feel is important.

Thank you for taking the time to participate in this study. I wanted remind you that you may choose not to answer any of the questions and can end your participation at any time during this interview. Your confidentiality will be respected and your name will not be associated with the data. We will be audio-recording this session. Please let me know if there are any questions before we begin.

8. Can you tell me a little bit about yourself?
    a. How long have you been a xxx (physio, physician, etc)
    b. How long have you been in the primary care setting?
    c. Can you give me a brief description about the type of clinic you work at?
        i. Are there other health care professionals present? Solo?

9. Can you please tell me how you would describe frailty?

10. Can you tell me a bit about your experience with older adults living with or at risk for frailty?
    a. What are your interactions like?
    b. How often do you see frail older adults within a typical work week?

11. What kinds of concerns do you have for older adults at risk for, or living with frailty?
12. Can you describe how you would currently assess patient’s frailty status?
    a. Probe: what tools do you currently utilize?
        i. If clinical judgement: what does this mean?

13. What kinds of indicators do you look for that would suggest a person is at risk?
    a. Probe: what initiates a frailty assessment?

14. Do you think that screening tools encompass the areas that influence frailty?
    a. Can you tell me why you feel this way?

15. Are there any gaps or limitations in screening tools that you currently use or know of?
    a. Do you feel there are components that should be added?

16. Are there specific measures or indicators that you choose to monitor that are not part of a formal frailty screening assessment?
    a. Why do you monitor these?

17. Do your older patients voice concerns over their psychological or social well-being?
    a. Yes: how do they voice these concerns?
    b. No: Why do you think they do not?

18. Is there anything that I haven't asked about that you feel is important to share regarding frailty and psychosocial factors?
